# Associations between ambient air pollution and cancer incidence in Taiwan: an ecological study of geographical variations

**DOI:** 10.1186/s12889-019-7849-z

**Published:** 2019-11-09

**Authors:** Shih-Yung Su, Yung-Po Liaw, Jing-Rong Jhuang, Shu-Yi Hsu, Chun-Ju Chiang, Ya-Wen Yang, Wen-Chung Lee

**Affiliations:** 1 Institute of Epidemiology and Preventive Medicine, College of Public Health, National Taiwan University, Taipei, Taiwan; 2 Innovation and Policy Center for Population Health and Sustainable Environment, College of Public Health, National Taiwan University, Taipei, Taiwan; 3 Department of Public Health and Institute of Public Health, Chung Shan Medical University, Taichung, Taiwan; 4 Taiwan Cancer Registry, Taipei, Taiwan

**Keywords:** Cancer, Incidence, Air pollution, Kriging method, Correlation, Geographical variation

## Abstract

**Background:**

Air pollution is a global public health concern. The World Health Organization has recently set up a goal of saving 7 million people globally by 2030 from air pollution related death. We conducted an ecological study of geographical variation to explore the association between air pollution (specifically, particulate matter <2.5 μm in aerodynamic diameter [PM_2.5_], particulate matter <10 μm in aerodynamic diameter, sulfur dioxide, nitrogen dioxide, nitric oxide, and ozone) and cancer incidence in Taiwan, from 2012 to 2016.

**Methods:**

In this study, the yearly average concentrations of each air pollutant at 75 air quality monitoring stations were calculated, and using the kriging method, the concentrations were extrapolated to each and every geographical central point of 349 local administrative areas of Taiwan. Spearman rank correlation coefficients between the age-adjusted cancer incidence rates and various air pollutants were calculated by stratifying genders and urbanization degrees of the local administrative areas. A total of 70 correlation coefficients were calculated.

**Results:**

In total, 17 correlation coefficients were significantly positive at an alpha level of 0.05. Among these, four correlation coefficients between the age-adjusted cancer incidence rates and PM_2.5_ levels remained significant after Bonferroni correction. For men in developing towns, general towns, and aged towns and for women in aged towns, the age-adjusted cancer incidence rates increased 13.1 (95% confidence interval [CI], 8.8–17.6), 11 (95% CI, 5.6–16.4), 16.7 (95% CI, 6.9–26.4), and 11.9 (95% CI, 5.6–18.2) per 100,000 populations, respectively, for every 1 μg/m^3^ increment in PM_2.5_ concentrations.

**Conclusions:**

A significantly positive correlation was observed between the PM_2.5_ level and cancer incidence rate after multiple testing correction.

## Background

Air pollution is a major public health concern. In 2005, the World Health Organization (WHO) announced air quality guidelines for outdoor air pollution, including annual and daily permissible levels for fine particulate matter (particulate matter <2.5 μm in aerodynamic diameter [PM_2.5_]), coarse particulate matter (particulate matter <10 μm in aerodynamic diameter [PM_10_]), sulfur dioxide (SO_2_), nitrogen dioxide (NO_2_), and ozone (O_3_) [1]. In 2018, WHO hosted the first conference on air pollution and health, and set up a goal of saving 7 million people globally by 2030 from death due to air pollution [2].

Air pollution is associated with cardiovascular diseases [3], chronic obstructive pulmonary disease [4], and specific types of cancers, such as oral, lung, breast, liver, bladder, kidney, prostate and ovarian [5–18], and is estimated to cause 4.2 million premature deaths globally [19]. Moreover, outdoor air pollution has been classified as a Group 1 carcinogen (carcinogenic to humans) by the International Agency for Research on Cancer (IARC) [20].

Through an ecological study, the association between air pollution and disease morbidity can be conveniently explored, and the results can be used to establish measures to quickly respond to the urgent public health concerns. We took an integrated approach and conducted an ecological study of geographical variation to explore the association between air pollution and incidence of all types of cancer in Taiwan.

## Methods

All incident cancer cases from 2012 to 2016 were extracted from the Taiwan Cancer Registry database. Cancer cases were categorized based on gender, age (seven age groups: 20–29, 30–39, 40–49, 50–59, 60–69, 70–79, and ≥ 80 years), and residence (a total of 349 local administrative areas [LAAs], excluding the 19 LAAs in offshore islands). We excluded cases in which patients aged less than 20 years because of the paucity of data. Population numbers from 2012 to 2016 were extracted from an online database provided by the Department of Statistics of the Ministry of the Interior in Taiwan and were similarly classified. Because the age structures of the LAAs are disparate, the age-adjusted cancer incidence rates were calculated using the 2000 World Standard Population proportions from WHO.

Air pollution monitoring data from 2012 to 2016 were provided by the Environmental Protection Administration of Taiwan. A total of 75 air quality monitoring stations (all located in the main island of Taiwan) monitored hourly concentrations of PM_2.5_, PM_10_, SO_2_, nitric oxide, NO_2_, and O_3_. However, we calculated the coarser yearly average concentrations of each air pollutant at these monitoring stations because the data of these stations may be lacking for several months during a year due to regular maintenance. Subsequently, the kriging method [21] was used to extrapolate these concentrations, based on the spatial correlations between air quality monitoring stations, to each and every geographical central point of all 349 LAAs (the longitudes and latitudes of which were taken from the online data provided by the National Land Surveying and Mapping Center of the Ministry of the Interior in Taiwan). Gaussian, exponential, Matérn, spherical, cubic, pentaspherical, and hole-effect models were fit to the semivariograms (a spatial correlation function of the distance between stations), constructed for station pairs with distances less than 50 km (a total of 640 pairs), and the best-fit model was selected (the smallest sum of squared errors). Ordinary kriging equations were used for interpolations. We assumed that the exposure levels of outdoor air pollution for people from the same LAA were the same for all age groups.

We calculated Spearman rank correlation coefficients between the age-adjusted cancer incidence rates and various air pollutants by stratifying genders and urbanization degrees of the LAAs. Gender stratification should reduce some of the confounding effects of tobacco smoking as the smoking prevalence in Taiwan is strongly gender-dependent (Additional file 1: Table S1). The urbanization degree was defined based on demographic characteristics (such as population density, education level, and the proportion of elderly people), industrialization, and medical resources in each LAA. A total of seven urbanization degrees were defined: metropolises, cities, developing towns, general towns, aged towns, agriculture towns, and villages [22]. A total of 70 hypotheses were tested, and Bonferroni correction was used for counteracting the multiple testing problem. All statistical analyses (including the kriging method) were conducted using SAS software 9.4 version (SAS® Institute Inc., Cary, North Carolina, USA). QGIS software 3.6 version (QGIS Development Team, n.d.) was used to draw maps.

This study was approved by the Data Release Review Board of the Health Promotion Administration, Ministry of Health and Welfare in Taiwan, which waived the requirement for informed consent.

## Results

**Fig. 1:**
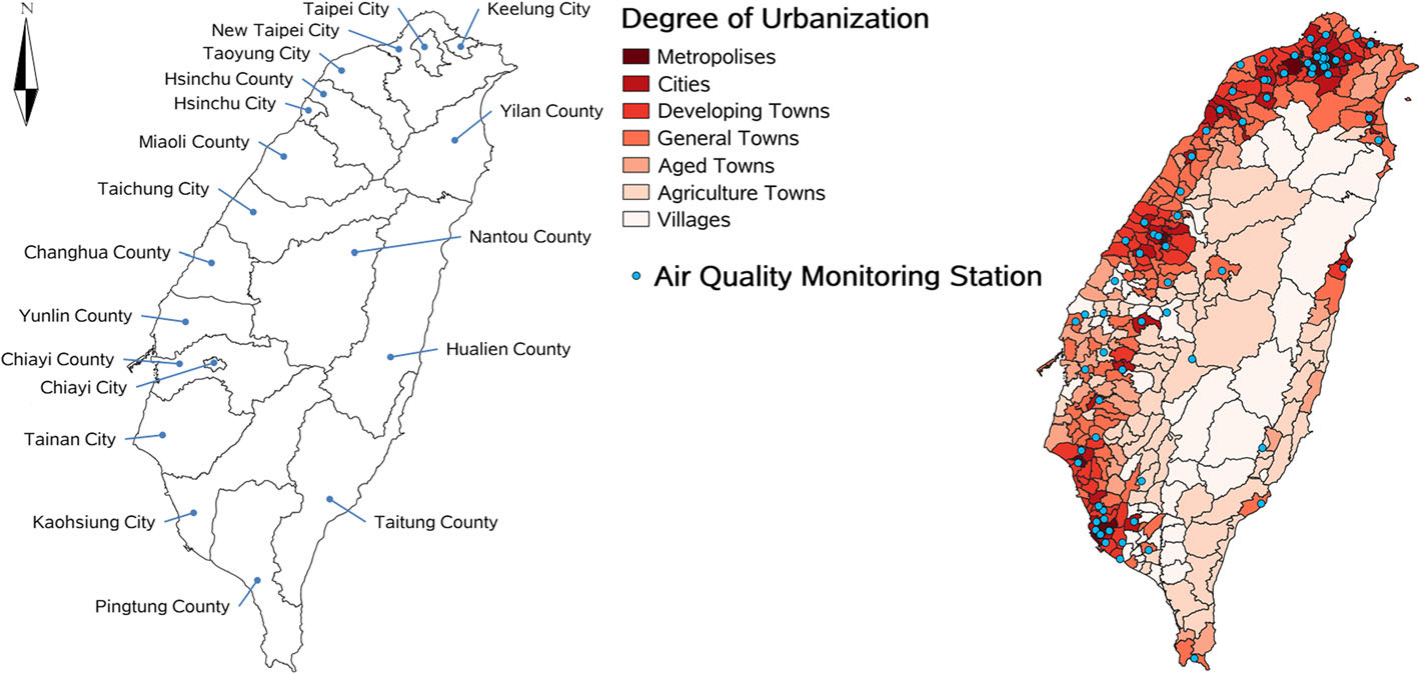
The 349 local administrative areas, 75 monitoring stations and 7 degrees of urbanization

Nineteen cities/counties in the main island of Taiwan are listed in Fig. 1. The same figure also presents the 349 LAAs (classified into seven urbanization degrees) and the 75 air quality monitoring stations. Among the 349 LAAs, 27 LAAs were metropolises (7.7%), 42 were cities (12%), 57 were developing towns (16.3%), 85 were general towns (24.4%), 31 were aged towns (8.9%), 60 were agricultural towns (17.2%), and 47 were villages (13.5%). The metropolises and cities were mainly located in northern and western Taiwan, whereas agricultural towns and villages were located in the central mountain range. The air quality monitoring stations were distributed throughout the main island, but were concentrated in northern and western Taiwan.

**Fig. 2:**
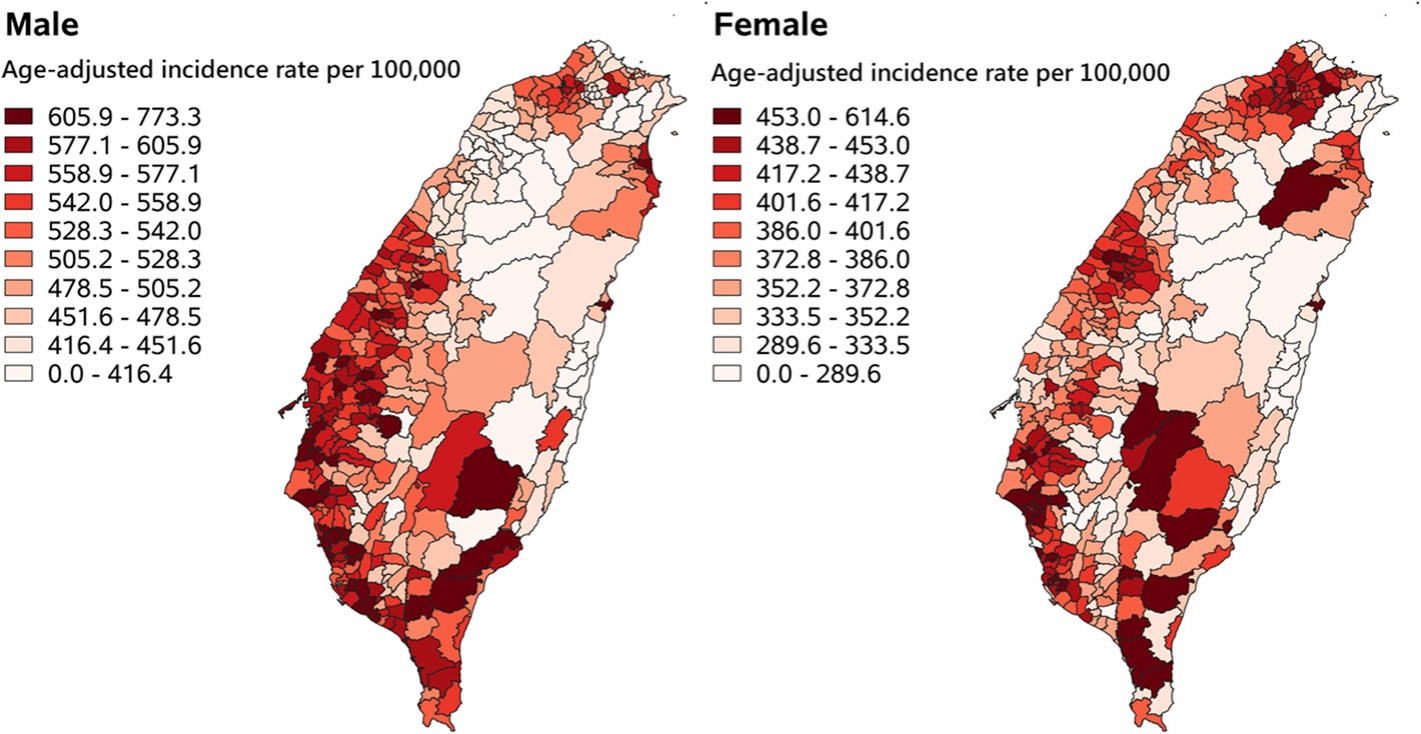
Cancer map. Decile is used for classification of pattern

The age-adjusted cancer incidence rates for men and women from 2012 to 2016 in the 349 LAAs are presented in Fig. 2. For men, the age-adjusted rates in 82.2% of the LAAs in southwestern and midwestern Taiwan were higher than 528.3 (the median of Taiwan) per 100,000 population. Eleven LAAs in Kaohsiung City, five in Tainan City, four in Pingtung County, four in Yunlin County, and three in Chiayi County had the highest (top 10%) age-adjusted rates. For women, the LAAs with the highest age-adjusted rates were located in southwestern (11 LAAs in Tainan City, 7 in Kaohsiung City, 4 in Pingtung County, and 1 in Chiayi County), midwestern (4 LAAs in Taichung City), and northern (4 LAAs in New Taipei City) Taiwan.

The distribution of yearly air pollution concentrations at the 75 air quality monitoring stations from 2012 to 2016 is presented in Table 1. The mean (minimum and maximum) concentrations of PM_2.5_, PM_10_, SO_2_, NO_x_ (the combination of NO and NO_2_), and O_3_ were 25.2 (6.3 and 45.4) μg/m^3^, 49.5 (15.7 and 81.9) μg/m^3^, 3.3 (1.2 and 8.9) ppb, 20.5 (2.4 and 116.8) ppb, and 29 (16.7 and 42.8) ppb, respectively.

**Table 1:** Distribution of air pollution concentration from 2012 to 2016

Air pollutant (units)	Mean (SD)	Minimum	Percentiles					Maximum
			5th	25th	50th	75th	95th	
PM_2.5_ (μg/m^3^)	25.182 (7.502)	6.280	12.297	20.189	25.109	29.803	39.255	45.380
PM_10_ (μg/m^3^)	49.500 (13.615)	15.690	28.400	39.316	48.454	59.708	72.443	81.931
SO_2_ (ppb)	3.338 (1.237)	1.203	1.715	2.626	3.102	3.828	6.037	8.861
NO_x_ (ppb)	20.470 (14.044)	2.374	7.749	13.601	17.726	23.215	37.012	116.787
O_3_ (ppb)	28.952 (3.726)	16.677	23.026	26.729	28.734	31.053	36.035	42.777

SD indicates standard deviation

NO_x_ refers to the combination of NO and NO_2_

**Fig. 3:**
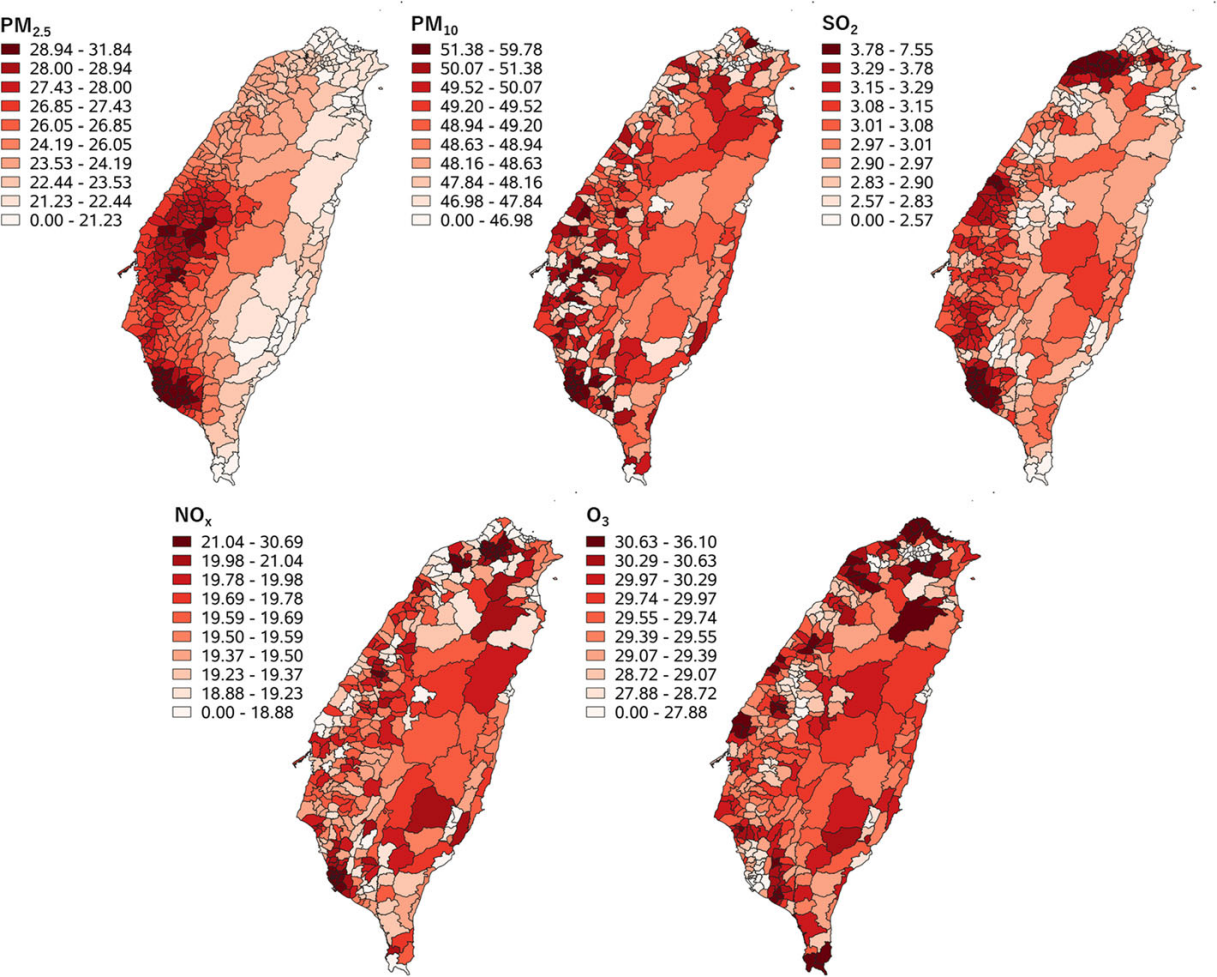
Air pollution map. Decile is used for classification of pattern

Figure 3 presents the predicted (kriging interpolated) air pollution concentrations for the 349 LAAs (kriging analysis details are presented in Additional file 1: Appendix 1, including the best-fit models and the estimated values of the ranges, sills, and nuggets for the various pollutants). Two major clusters of high PM_2.5_ levels were found in midwestern (5 LAAs in Yunlin County and 3 LAAs in Chiayi County) and southern (19 LAAs in Kaohsiung City and 6 LAAs in Pingtung County) Taiwan. Furthermore, the LAAs with high PM_10_ levels were widely distributed over southwestern Taiwan. In addition, three clusters of high SO_2_ levels were found in northern (8 LAAs in Taoyuan City and 4 LAAs in New Taipei City), midwestern (3 LAAs in Changhua County), and southern (18 LAAs in Kaohsiung City and 2 LAAs in Pingtung County) Taiwan. Moreover, two small clusters of high NO_x_ levels were detected in northern (9 LAAs in Taipei City, 8 in New Taipei City, and 3 in Taoyuan City) and southern (12 LAAs in Kaohsiung City) Taiwan. Finally, LAAs with high O_3_ levels were found to be distributed across the main island of Taiwan.

Table 2 presents Spearman rank correlation coefficients between the age-adjusted cancer incidence rates and various air pollutants. A total of 17 correlation coefficients were significantly positive at an alpha level of 0.05. Among these, the following four correlation coefficients between the age-adjusted cancer incidence rates and PM_2.5_ levels remained significant after Bonferroni correction: the correlation coefficient of 0.60 in developing towns, 0.46 in general towns, 0.66 in aged towns for men, and 0.63 in aged towns for women.

**Table 2:** Spearman’s rank correlation coefficients between the age-adjusted cancer incidence rate and the various air pollutants by gender and urbanization degrees

	Men					Women				
	PM_2.5_	PM_10_	SO_2_	NO_x_	O_3_	PM_2.5_	PM_10_	SO_2_	NO_x_	O_3_
Metropolises	0.457^a^	0.276	0.515^a^	−0.326	0.465^a^	0.214	0.236	−0.037	−0.344	0.542^a^
Cities	0.280	0.327^a^	0.300	0.083	−0.210	0.132	0.195	0.163	0.131	−0.050
Developing towns	0.599^b^	0.340^a^	0.022	0.334^a^	−0.136	0.244	0.285^a^	−0.025	0.356^a^	0.070
General towns	0.450^b^	−0.011	0.340^a^	0.013	−0.151	0.248^a^	−0.099	0.236^a^	−0.022	−0.128
Aged towns	0.664^b^	0.144	0.179	−0.102	0.225	0.634^b^	−0.067	0.305	−0.235	0.336
Agricultural towns	0.247	−0.147	0.211	−0.240	−0.138	0.122	−0.017	0.211	−0.181	0.028
Villages	0.371^a^	−0.050	0.214	0.015	0.106	−0.218	−0.002	− 0.043	0.029	− 0.094

^a^indicates significance at an alpha level of 0.05

^b^indicates significance at the Bonferroni-corrected alpha level of 0.000714 (a total of 70 hypothesis tests being performed)

**Fig. 4:**
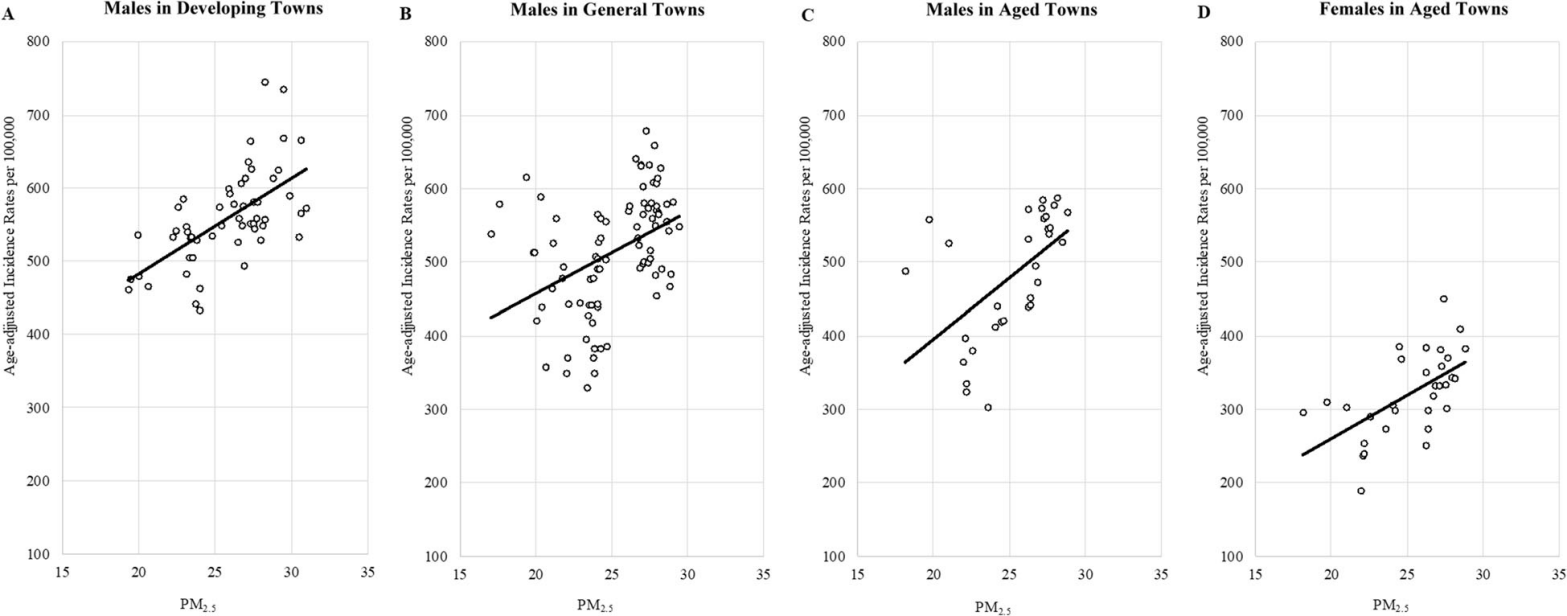
Dose-response relationship of age-adjusted cancer incidence and fine particulate matter in developing towns (panel A), general towns (panel B) and aged towns (panel C) for males and aged towns (panel D) for females

Figure 4 presents the dose–response relationships of the Bonferroni-corrected significant associations. The age-adjusted cancer incidence rates for men in developing towns, general towns, and aged towns increased 13.1 (95% confidence interval [CI], 8.8–17.6), 11 (95% CI, 5.6–16.4), and 16.7 (95% CI, 6.9–26.4) per 100,000 population, respectively, for every 1 μg/m^3^ increment in the PM_2.5_ concentration. In addition, the age-adjusted rate for women in aged towns increased 11.9 (95% CI, 5.6–18.2) per 100,000 population for every 1 μg/m^3^ increase in the PM_2.5_ concentration. Furthermore, the dose–response relationships for other significant associations at an alpha level of 0.05 (but not after Bonferroni correction) are presented in Additional file 1: Figure S1.

## Discussion

This geographical association study revealed positive correlations between PM_2.5_ levels and age-adjusted cancer incidence rates, which remained significant after rigorous correction of multiple comparisons. PM_2.5_ can penetrate deeply into the lungs and other organs of the respiratory system [4]. According to WHO air quality guidelines, more than 95% of the monitored concentrations were higher than the annual permissible levels (10 μg/m^3^) in Taiwan. Moreover, according to the air quality criteria established by the Environmental Protection Administration in Taiwan in 2012, more than 75% of the monitored concentrations for the study period exceeded the annual permissible levels (15 μg/m^3^).

In Taiwan, the anthropogenic sources of PM_2.5_ include folk activities (incense, crop, firecracker, and firework burning), industrial activities (power plant, petrochemical, and nonpetrochemical), fugitive dust from roads and construction works, and traffic-related exhaust emissions [23], and the natural sources of PM_2.5_ include wildfires and river fugitive dust [24]. To understand the composition of air pollution, the Taiwanese government has implemented the Taiwan Emission Data System (TEDS) since 1992. According to a recent report of TEDS, the major sources of PM_2.5_ were fugitive dust from roads and construction works, traffic-related exhaust emissions, and industrial emissions (all anthropogenic emissions), which accounted for 41, 23, and 22%, respectively, of the total emissions [25]. For the two clusters of PM_2.5_ (Fig. 3), 93, 90, 86, and 81% of the total emissions in Kaohsiung City, Yunlin County, Chiayi County, and Pingtung County were attributable to such anthropogenic emissions [25].

Our findings have added to the growing evidence of the negative consequence of PM_2.5_ on health. A recent report by IARC indicated that polluted air is a complex mixture of carcinogenic and mutagenic substances such as cigarette smoke and can widely affect the lungs and other organs [26, 27]. Exposure to PM_2.5_ can lead to inflammatory injury, immune response stress, oxidative DNA damage, DNA methylation, and insufficient DNA repair [28–32]. Such genotoxic effects may increase cancer risk [33].

Recent observational studies have revealed the harmful effect of PM_2.5_ on different cancer sites. According to a cohort study in the United States exploring the association of PM_2.5_ with mortality from 29 cancer sites, bladder and kidney cancers had significantly positive associations [5]. Another cohort study found that exposure to PM_2.5_ may increase liver cancer incidence in Europeans (although not statistically significant) [6]. Furthermore, previous studies in Taiwan have revealed that exposure to high PM_2.5_ levels was significantly associated with increased risks of lung, liver, breast, oral, and ovarian cancer [7–12]. We further examined the associations of PM_2.5_ with cancer types in developing, general, and aged towns and found significantly positive correlations with oral, colorectal, liver, and skin cancers (Additional file 1: Table S2). However, the negative correlation coefficients of gastric and renal cancers were significant after Bonferroni correction.

Aside from PM_2.5_, study also indicated significantly positive correlations (but not after Bonferroni correction) between other air pollutants (PM_10_, SO_2_, NO_x_, and O_3_) and age-adjusted cancer incidence rates. According to the report of TEDS, 93 and 89% of the total emissions for PM_10_ and NO_x_, respectively, were attributable to fugitive dust from roads and construction works, traffic-related exhaust emissions, and industrial emissions [25] (TEDS does not provide SO_2_ and O_3_ estimates). Studies have also suggested that air pollution due to pollutants other than PM_2.5_ is associated with an increased risk of lung, breast, prostate, and ovarian cancers [13–18].

We categorized each air pollutant into quartiles and calculated the population attributable fractions (PAFs) with the first (lowest) quartile as the baseline (Additional file 1: Table S3). The PAF due to PM_2.5_ was the largest; a total of 6.8% of cancer incidences can be attributable to high PM_2.5_ levels. Using the rate ratio estimates presented in Additional file 1: Table S3, we found that the PAFs due to PM_2.5_ were similar for men and women, and for the older (age, ≥60 years) and young (age, <60 years) individuals. The PAFs due to PM_2.5_ were higher in rural areas (aged towns, agricultural towns, and villages) than in urban areas (metropolises, cities, developing towns, and general towns), 8.2% versus 6.7%. For the geographical variations, the PAFs due to PM_2.5_ were 9.8, 8.3, 4.3, and 3.6%, respectively, for the southern, western, northern, and eastern parts of Taiwan. However, the aforementioned analyses assumed that the lag time between the exposures of air pollution and cancer incidence did not confound the estimations.

Reducing ambient air pollution is a control target of the sustainable development goals (SDGs) [34]. To meet the SDG of reducing air pollution caused by traffic exhaust emissions, planning a well-conceived transport strategy, reducing traffic congestion, and improving public transport networks are suggested. In Taiwan, a high correlation between PM_2.5_ concentrations and automobile usage was noted [7]. In December 2018, approximately 14 million motorcycles and 8 million cars (i.e., 58.6 motorcycles and 34.1 cars per 100 people, respectively) were registered in the Ministry of Transportation and Communications in Taiwan [35]. The Taiwanese government has implemented a policy of green incentives since 2009 to increase electric motorcycle production. In 2017, the market share of electric motorcycles manufactured in Taiwan accounted for 5.3% of global sales [36]. Another target of SDGs was to reduce the mortalities due to noncommunicable diseases (NCDs) by one-third. Cancer is one of the major NCDs. In this study, the highest cancer incidence rates were found in southwestern and midwestern Taiwan and were also significantly related to high PM_2.5_ levels. Therefore, reducing air pollution in such areas should be prioritized.

A number of limitations may compromise the study results. First, we standardized and stratified data to adjust for confounding effects due to age, gender, and urbanization. Possible confounding effects due to tobacco smoking, however, could not be adjusted for because of the lack of access to data. The Adult Smoking Behavior Survey implemented by the Ministry of Health and Welfare in Taiwan has investigated and collected the yearly cross-sectional data of smoking prevalence since 1997; however, the results were not detailed down to each LAA. Second, the air quality monitoring stations in Taiwan are purposely constructed near emission sources, such as densely populated areas, and downwind of industrial districts and dynamic traffic environments; however, all emission sources might not be covered by the monitoring stations, resulting in selection biases. Additional studies are needed to adjust for the confounding effect of smoking, and they should use a more accurate model, such as land-use regression [37], to clarify the association between ambient PM_2.5_ concentrations and cancer risk. Third, the five-year average cancer incidence rates were calculated to reduce random errors because many LAAs have small population sizes. Several LAAs in the central mountain range (Haiduan, Taoyuan, Yanping, Alishan, and Namasia) presented unusually high cancer incidence rates, possibly due to random errors resulting from the extremely small population size of these LAAs. Finally, this was an exploratory study based on aggregated data. An individual-level information such as body mass index, glycosylated hemoglobin, blood pressure, drug use, dietary pattern, and lifestyles is required for a finer confounding control. Further studies should combine the aggregated and the individual-level data in a hierarchical model to better clarify the associations between air pollutants and cancers.

## Conclusions

The highest age-adjusted cancer incidence rates for both sexes and the hotspots of high PM_2.5_ levels were all located in southwestern and midwestern Taiwan. This geographical association study revealed significant positive correlations between PM_2.5_ concentrations and age-adjusted cancer incidence rates after multiple testing correction. Positive correlations for other air pollutants (i.e., PM_10_, SO_2_, NO_x_, and O_3_) were also found, but not after Bonferroni correction. Additional studies are required to confirm or refute these findings.

## Supplementary information

**Additional file 1: Appendix 1.** Details of the kriging analysis. **Figure S1.** The dose-response relationships for other significant associations under an alpha level of 0.05 but not after Bonferroni correction. **Table S1.** The smoking prevalence (%) in Taiwan. **Table S2.** Further analysis of spearman correlation between PM_2.5_ and 21 cancer sites. **Table S3.** Population attributable fractions due to air pollutants.

## Data Availability

The Taiwan Cancer Registry database is only available if any research institute has obtained permissions from the Department of Statistics, Ministry of Health and Welfare in Taiwan. For the cancer specific indices of the Taiwan Cancer Registry database, please refer to the link: https://cris.hpa.gov.tw/ (traditional Chinese only). For the application of the Taiwan Cancer Registry database usage, please contact the corresponding author (e-mail: wenchung@ntu.edu.tw) of this paper for more information.

## Abbreviations

ASBS: Adult smoking behavior survey
EPA: Environmental protection administration
IARC: International agency for research on cancer
LAAs: Local administrative areas
NO_2_: Nitrogen dioxide
O_3_: Ozone
PM_10_: Coarse particulate matter
PM_2.5_: Fine particulate matter
SDGs: Sustainable Development Goals
SO_2_: Sulphur dioxide
TEDS: Taiwan Emission Data System
WHO: World Health Organization
