# Supplementary material for: Associations between ambient air pollution and cancer incidence in Taiwan: an ecological study of geographical variations
Source: BMC Public Health. 2019 Nov 9;19:1496. doi: 10.1186/s12889-019-7849-z (PMC6842529; doi:10.1186/s12889-019-7849-z)
Supplement: Supplementary file 1 — Additional file 1: Appendix 1. Details of the kriging analysis. Figure S1. The dose-response relationships for other significant associations under an alpha level of 0.05 but not after Bonferroni correction. Table S1. The smoking prevalence (%) in Taiwan. Table S2. Further analysis of spearman correlation between PM2.5 and 21 cancer sites. Table S3. Population attributable fractions due to air pollutants. [file 12889_2019_7849_MOESM1_ESM.docx]

**Appendix 1.** Details of the kriging analysis

The selected models to the semivariograms of PM_2.5_ are cubic model in 2012, hole-effect model in 2013 and 2015, and spherical model in 2014 and 2016. The estimations of range, sill and nugget are 17.76, 5.65 and 11.59 in 2012, 11.63, 8.96 and 9.05 in 2013, 48, 5.09 and 4.50 in 2014, 55.72, 5.73 and 5.68 in 2015, and 0.78, 0.34 and 1.35 in 2016.

For PM_10_, the selected models are hole-effect model in 2012 and 2013, and cubic model in 2014, 2015 and 2016. The estimations of range, sill and nugget are 7.93, 38.73 and 30.88 in 2012, 7.74, 40.73 and 40.16 in 2013, 2.63, 78.24 and 0 in 2014, 2.41, 59.02 and 0 in 2015, and 2.20, 56.21 and 0 in 2016.

For SO_2_, the selected models are cubic model in 2012, Matérn model in 2013 and 2014, and holeeffect model in 2015 and 2016. The estimations of range, sill and nugget are 28.74, 1.54 and 0.13 in 2012, 14.84, 1.63 and 0.02 in 2013, 13.31, 1.03 and 0.11 in 2014, 11.17, 0.59 and 0.11 in 2015, and 11.16, 0.76 and 0.06 in 2016.

For NO, the selected models are spherical model during 2012- 2016. The estimations of range, sill and nugget are 0, 98.98 and 47.74 in 2012, 0, 78.26 and 45.19 in 2013, 0, 72.90 and 44.11 in 2014, 0, 48.30 and 41.05 in 2015, and 0, 56.64 and 39.36 in 2016.

For NO_2_, the selected models are cubic model in 2012 and hole-effect model during 2013-2016. The estimations of range, sill and nugget are 1.98, 41.97 and 0 in 2012, 7.37, 24.09 and 18.16 in 2013, 10.87, 13.40 and 27.64 in 2014, 3.06, 29.53 and 4.70 in 2015, and 10.99, 13.81 and 20.10 in 2016.

For O_3_, the selected models are hole-effect model in 2012, 2013, 2014 and 2016, and cubic model in 2015. The estimations of range, sill and nugget are 13.43, 10.90 and 3.47 in 2012, 12.67, 8.64 and 5.49 in 2013, 11.39, 9.53 and 6.42 in 2014, 19.84, 1.16 and 14.34 in 2015, and 12.74, 9.31 and 2.84 in 2016.


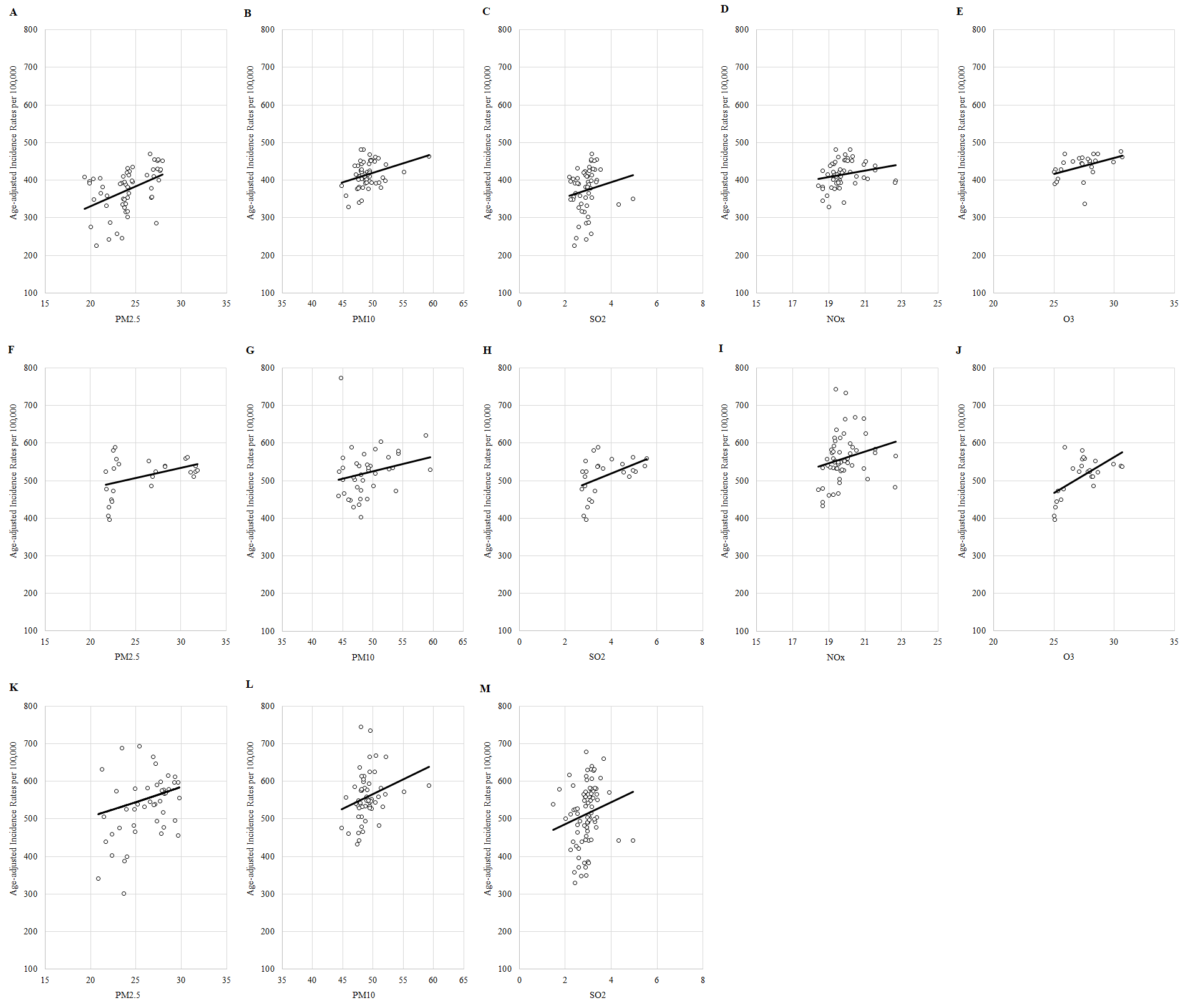


**Figure S1. The dose-response relationships for other significant associations under an alpha level of 0.05 but not after Bonferroni correction.** (Panel A: Females in ordinary town; Panel B: Females in new town; Panel C: Females in ordinary town; Panel D: Females in new town; Panel E: Females in high urbanization; Panel F: Males in high urbanization; Panel G: Males in middle urbanization; Panel H: Males in high urbanization;

Panel I: Males in new town; Panel J: Males in high urbanization; Panel K: Males in remote town; Panel L: Males in new town; Panel M: Males in ordinary town)

**Table S1. The smoking prevalence (%) in Taiwan**

|  | Men | | | | |  | Women | | | | |
| --- | --- | --- | --- | --- | --- | --- | --- | --- | --- | --- | --- |
|  | 2012 | 2013 | 2014 | 2015 | 2016 |  | 2012 | 2013 | 2014 | 2015 | 2016 |
| Taipei City | 25.5 | 35.2 | 23.7 | 24.9 | 22.9 |  | 5.8 | 3.3 | 2.8 | 5.7 | 4.2 |
| New Taipei City | 32.0 | 38.3 | 31.9 | 29.2 | 39.1 |  | 7.4 | 3.7 | 5.5 | 6.8 | 6.1 |
| Keelung City | 34.0 | 32.6 | 38.4 | 36.7 | 37.9 |  | 9.2 | 8.0 | 5.8 | 6.7 | 3.6 |
| Yilan County | 35.1 | 27.1 | 28.7 | 24.1 | 25.9 |  | 2.8 | 8.2 | 1.8 | 2.0 | 3.1 |
| Taoyung City | 30.7 | 35.6 | 35.3 | 34.3 | 21.8 |  | 6.8 | 2.9 | 2.2 | 6.4 | 3.8 |
| Hsinchu County | 34.3 | 25.5 | 27.8 | 32.8 | 28.0 |  | 3.9 | 0.7 | 5.1 | 1.9 | 3.6 |
| Hsinchu City | 30.9 | 24.7 | 25.7 | 22.6 | 35.4 |  | 2.7 | 3.3 | 6.0 | 4.5 | 5.9 |
| Miaoli County | 39.1 | 29.9 | 35.4 | 38.7 | 31.7 |  | 2.4 | 3.9 | 1.9 | 2.5 | 2.9 |
| Taichung City | 36.3 | 32.3 | 31.0 | 35.6 | 28.8 |  | 3.1 | 2.2 | 4.0 | 3.1 | 2.3 |
| Changhua County | 27.4 | 31.1 | 29.4 | 31.5 | 29.0 |  | 2.1 | 1.4 | 2.6 | 0.8 | 5.1 |
| Nantou County | 38.8 | 28.7 | 31.3 | 37.7 | 40.3 |  | 6.5 | 3.6 | 2.2 | 2.4 | 3.7 |
| Yunlin County | 31.6 | 33.9 | 30.7 | 28.2 | 35.9 |  | 2.3 | 0.9 | 1.0 | 1.4 | 3.8 |
| Chiayi County | 35.5 | 31.1 | 24.2 | 28.9 | 29.5 |  | 1.0 | 3.4 | 2.0 | 1.9 | 0.3 |
| Chiayi City | 33.0 | 30.2 | 31.2 | 21.8 | 30.0 |  | 2.2 | 1.6 | 2.9 | 2.4 | 2.8 |
| Tainan City | 26.5 | 26.9 | 29.9 | 26.8 | 27.3 |  | 2.0 | 2.2 | 2.9 | 3.3 | 2.4 |
| Kaohsiung City | 33.3 | 31.9 | 24.8 | 25.8 | 21.1 |  | 3.8 | 5.3 | 2.5 | 4.3 | 3.4 |
| Pingtung County | 30.9 | 31.2 | 26.0 | 34.2 | 19.7 |  | 4.6 | 2.6 | 1.3 | 1.6 | 3.3 |
| Hualien County | 33.5 | 30.1 | 32.9 | 26.6 | 28.7 |  | 3.3 | 4.6 | 12.5 | 3.0 | 2.3 |
| Taitung County | 31.9 | 31.2 | 29.6 | 31.5 | 24.1 |  | 9.5 | 5.4 | 4.5 | 11.4 | 6.1 |

**Table S2. Further analysis of spearman correlation between PM_2.5_ and 21 cancer sites**

|  | Men | | |  | Women |
| --- | --- | --- | --- | --- | --- |
|  | Developing  towns | General  towns | Aged  towns |  | Aged  towns |
| Oral | 0.591** | 0.557** | 0.588** |  | -0.329* |
| Oropharynx and Hypopharynx | 0.229 | 0.328* | -0.032 |  | -0.241 |
| Nasopharynx | 0.200 | 0.090 | -0.294* |  | -0.343* |
| Esophagus | -0.074 | 0.167 | -0.217* |  | -0.295* |
| Stomach | -0.583** | -0.292* | -0.451** |  | -0.425** |
| Colon and Rectum | 0.334* | 0.330* | 0.454** |  | 0.089 |
| Liver | 0.424* | 0.690** | 0.427** |  | 0.162 |
| Pancreas | -0.197 | 0.262 | -0.136 |  | -0.283* |
| Lung | -0.099 | 0.091 | 0.174 |  | 0.175 |
| Skin | 0.509* | 0.599** | 0.286* |  | 0.411** |
| Breast |  |  |  |  | 0.204 |
| Cervix |  |  |  |  | -0.184 |
| Corpus Uteri |  |  |  |  | -0.093 |
| Ovary |  |  |  |  | -0.170 |
| Prostate | -0.141 | 0.207 | 0.181 |  |  |
| Bladder | -0.079 | 0.183 | 0.098 |  | 0.106 |
| Kidney | -0.567** | -0.144 | -0.295* |  | -0.295* |
| Brain | 0.092 | -0.005 | -0.166 |  | 0.005 |
| Thyroid | 0.099 | 0.031 | 0.102 |  | 0.108 |
| Leukemia | -0.133 | -0.284* | -0.170 |  | -0.247* |
| Non-Hodgkin Lymphoma | 0.237 | -0.059 | -0.340* |  | -0.052 |

* indicates significance at an alpha level of 0.05

**indicates significance at the Bonferroni-corrected alpha level of 0.000704 (a total of 71 hypothesis tests being performed)

**Table S3. Population attributable fractions due to air pollutants**

| Air pollutant (unit) | Person-year | Cases | Rate ratio (95% CIs) | | P-value | | | Population attributable fraction |
| --- | --- | --- | --- | --- | --- | --- | --- | --- |
| PM_2.5_ (μg/m^3^) |  |  |  | |  | | |  |
| <20.189 | 19,986,873 | 107,671 | Reference | | | | | |
| 20.189~25.109 | 18,652,349 | 102,764 | 1.06 (1.05 to 1.07) | | <0.001 | | | 1.15% |
| 25.109~29.803 | 39,891,769 | 221,242 | 1.09 (1.08 to 1.10) | | <0.001 | | | 3.71% |
| >29.803 | 13,156,339 | 74,590 | 1.14 (1.13 to 1.15) | | <0.001 | | | 1.98% |
| PM_10_ (μg/m^3^) |  |  |  |  | |  |  |  |
| <39.316 | 1,439,243 | 7,961 | Reference | | | | | |
| 39.316~48.454 | 48,742,452 | 269,091 | 1.00 (0.97 to 1.02) | | 0.7407 | | | - |
| 48.454~59.708 | 37,732,221 | 207,344 | 1.02 (0.99 to 1.04) | | 0.2086 | | | - |
| >59.708 | 3,773,414 | 21,871 | 1.07 (1.05 to 1.10) | | <0.001 | | | 0.30% |
| SO_2_ (ppb) |  |  |  |  | |  |  |  |
| <2.626 | 12,831,973 | 71,929 | Reference | | | | | |
| 2.626~3.102 | 31,911,793 | 174,938 | 1.00 (0.99 to 1.01) | | 0.535 | | | - |
| 3.102~3.828 | 28,902,047 | 161,970 | 1.04 (1.04 to 1.05) | | <0.001 | | | 1.39% |
| >3.828 | 18,041,517 | 97,430 | 1.06 (1.05 to 1.07) | | <0.001 | | | 1.12% |
| NO_x_ (ppb) |  |  |  |  | |  |  |  |
| <13.601 | 48,292 | 300 | Reference | | | | | |
| 13.601~17.726 | 7,725,569 | 43,790 | 0.92 (0.82 to 1.03) | | 0.1332 | | | - |
| 17.726~23.215 | 69,019,953 | 378,993 | 0.92 (0.82 to 1.03) | | 0.1456 | | | - |
| >23.215 | 14,893,516 | 83,184 | 0.92 (0.82 to 1.03) | | 0.1422 | | | - |
| O_3_ (ppb) |  |  |  |  | |  |  |  |
| <26.729 | 15,441,280 | 85,617 | Reference | | | | | |
| 26.729~28.734 | 25,926,906 | 142,577 | 1.04 (1.03 to 1.05) | | <0.001 | | | 1.00% |
| 28.734~31.053 | 40,501,131 | 224,346 | 1.02 (1.01 to 1.03) | | <0.001 | | | 1.03% |
| >31.053 | 9,818,013 | 53,727 | 1.00 (0.99 to 1.02) | | 0.4698 | | | 0.05% |

†Poisson regression models for five air pollutants, adjusting for sex, age groups, calendar years and urbanization degrees.
